# Gluten Proteins: Beneficial Factors and Toxic Triggers in Human Health

**DOI:** 10.3390/foods14193403

**Published:** 2025-10-01

**Authors:** Luigia Di Stasio, Gianfranco Mamone

**Affiliations:** Institute of Food Sciences, National Research Council, 83100 Avellino, Italy; luigia.distasio@isa.cnr.it

**Keywords:** celiac disease, non-celiac gluten sensitive, gluten hydrolysis approaches, gluten protein, bioactive peptides

## Abstract

The impact of gluten on human health has been the subject of intense study. Gluten proteins are implicated in a range of adverse health effects, such as allergy, celiac disease, and non-celiac gluten sensitivity in predisposed individuals. However, beyond their potential to trigger adverse reactions, gluten proteins also harbor sequences that, upon digestion or fermentation, can release bioactive peptides with health-promoting properties. These peptides have been reported to exhibit antioxidant, antihypertensive, and immunomodulatory activities, suggesting that gluten-derived products may contribute positively to human health. This review aims to explore the dual nature of gluten proteins, examining their role as both potential health hazards and sources of beneficial molecules. By dissecting the molecular mechanisms underlying gluten-related disorders and the functional properties of gluten-derived peptides, we seek to provide a balanced view of gluten’s complex role in nutrition and health.

## 1. Introduction

Gluten is a complex mixture of storage proteins predominantly found in the endosperm of wheat (*Triticum* species) and, to a lesser extent, in other cereals such as barley and rye. Because of its unique rheological and functional properties, gluten is a critical component in food processing, particularly in the production of pasta and bakery food products. Gluten also affects consumer acceptability, especially with regard to product texture and appearance. The quantity and quality of gluten proteins are key criteria in wheat breeding and flour quality assessment [1]. Given its central role in food formulation and processing, gluten remains a highly valued ingredient in the global food industry, despite growing health concerns among certain populations, since its consumption is also associated with a spectrum of adverse health effects in susceptible individuals. The most well-characterized condition is celiac disease (CD), an autoimmune enteropathy triggered by the ingestion of gluten peptides in genetically predisposed individuals. CD prevalence in the general population is estimated at approximately 1% [2]. Other gluten-related disorders include non-celiac gluten sensitivity (NCGS), which is characterized by gastrointestinal and extraintestinal symptoms in the absence of celiac-specific serology or histological findings, and wheat allergy, an IgE-mediated hypersensitivity reaction [3].

Despite these well-documented adverse effects, gluten proteins are also a source of peptides with potential health-promoting properties, which are released during the enzymatic hydrolysis process, such as those naturally occurring during gastrointestinal digestion, or during fermentation mediated by microbial proteases [4].

The contrasting roles of gluten, as both a health risk and a potential source of beneficial compounds, highlight the need for a comprehensive and evidence-based evaluation of its effects on human health. The recent ongoing research has focused on peptide identification, functional validation, and controlled food processing strategies, which are helpful to mitigate gluten-related risks while enhancing its positive contributions to nutrition and well-being [5].

In light of the duality that characterizes gluten proteins, being both potential health hazards and sources of beneficial molecules, this review aims to provide a comprehensive overview of their structural, functional, and biological properties. We first describe the molecular composition and classification of gluten proteins, with an emphasis on their unique physicochemical features relevant to food processing. We then explore the mechanisms underlying gluten-related disorders, including CD, NCGS, and wheat allergy, highlighting the specific peptide sequences and immune pathways involved. Finally, we examine emerging evidence on gluten-derived bioactive peptides and their potential health-promoting effects, focusing on their generation during digestion and fermentation.

By integrating insights from food science, immunology, and nutritional biochemistry, this review seeks to present a balanced and updated perspective on gluten, moving beyond its role as a dietary trigger of disease to consider its possible functional applications in human health.

## 2. Structure and Composition of Gluten Proteins

### 2.1. Classification and Structure of Gluten Proteins

According to the Osborne classification based on solubility in aqueous alcohols, gluten proteins can be classified into two main fractions: soluble-monomeric gliadins and the insoluble-polymeric glutenins [1]. Furthermore, based on electrophoretic mobility and molecular weights (MWs), gliadins are classified in four different types: ω5, ω1,2, α/β, and γ-gliadins, which have also been redefined based on their amino acid sequences as sulphur (S)-rich (α- and γ-type) and S-poor (ω-type) prolamins [6]. The gliadins ω5- and ω1,2-gliadins are characterized by a high content of glutamine, proline, and phenylalanine (but poor in cysteine), and their repetitive sequences distinguish almost entirely their protein structure (e.g., such as QQPQQPFP in ω1,2 gliadins and QQQFP in ω5-gliadins). While α/β and γ-gliadins are characterized by a much lower content of glutamine and proline than ω-gliadins, and their N-terminal and C-terminal profile are clearly distinct. Within the C-terminal domains, in fact, α/β- and γ-gliadins are almost homologous. Although the N-terminal domain, representing about 50% of the total structure, consists mostly of repetitive sequences rich in glutamine, proline, phenylalanine, and tyrosine (e.g., the dodecapeptides QPQPFPQQPYP, which are usually repeated five times in α/β- gliadins; QPQPFP and PQQPYP in α-gliadins, and the QPQQPFP in γ-gliadins, which is repeated up to 16 times) [7,8].

Glutenins, following the reduction of inter-chain and intra-chain disulfide bonds, consist of two main groups named high-molecular-weight (HMW)-GS and low-molecular-weight (LMW)-GS. LMW-GS can be further subdivided into B, C, and D-type, based on their SDS-PAGE mobility and isoelectric point (pI). While on the basis of N-terminal amino acid sequences, three subgroups of typical LMW-GS can be classified, called LMW-*s*, LMW-*m,* and LMW-*i* types, according to the first amino acid residue of the mature protein: serine, methionine, or isoleucine, respectively. HMW-GS are subdivided into *x*- and *y*-types, encoded by closely linked genes at the Glu-A1 and Glu-B1 loci. Structurally, HMW-GS consists of three domains characterized by a C-terminal domain, which contains cysteine residues involved in inter-chain and intra-chain disulfide bonds, a non-repetitive N-terminal domain, and a repetitive central domain that promotes intermolecular hydrogen bonding and that is characterized by repetitive units comprising mainly glutamine, glycine, and proline, such as PGQGQQ (both types), GYYPTSPQQ or GYYPTSLQQ, and GQQ (*x*-type), and HYPASQ (*y*-type) [6,7,8,9,10].

The formation of the gluten network during dough mixing involves a complex series of reactions and molecular interactions that specifically involve gliadins and glutenins. These proteins interact through both covalent and non-covalent bonds, which are essential for developing the viscoelastic properties of dough. Among the covalent interactions, disulfide bonds (S–S bridges) play a critical role [11]. These bonds are formed through the oxidation of cysteine residues present in the protein structure. In gliadins, which are monomeric proteins, disulfide bonds are predominantly intrachain, contributing to their extensibility. In contrast, glutenins, particularly the HMW subunits, form both intrachain and interchain disulfide bonds. The interchain disulfide bridges in glutenins are especially important, as they facilitate the formation of large, high-molecular-weight protein polymers that contribute significantly to the elasticity, strength, and overall viscoelasticity of the gluten network [12]. In particular, the molecular weight distribution of glutenins is a critical factor in determining dough quality, as it reflects the extent of disulfide bonding within the gluten network. This distribution is influenced by genetic factors (e.g., presence of specific HMW subunits like Dx5, the HMW-GS/LMW-GS ratio, and the abundance of chain terminators), environmental conditions (such as sulfur deficiency, heat, and water stress), and the redox environment during dough mixing, where reducing or oxidizing agents regulate disulfide bond formation and rearrangement. Together, these factors modulate gluten polymer size and structure, ultimately shaping dough functionality and rheological properties [13,14].

Additionally, non-covalent interactions such as hydrogen bonding—primarily due to the high glutamine content in gluten proteins—as well as intramolecular hydrogen bonds, hydrophobic interactions, Van der Waals forces, and ionic bonds, further stabilize the gluten structure and modulate its functional properties during mixing and baking [12].

### 2.2. Gluten-Related Proteins in Non-Wheat Cereals

The corresponding forms of gluten proteins in wheat (8–12% of flour) are also present in barley (~4%), rye (~3%), and oats (<2%), where they are called hordein, secalin, and avenin, respectively [15]. Due to the presence of celiac-active polypeptides, these proteins can trigger an immune-mediated inflammatory response in patients with CD [16,17].

Specifically, ω-secalins, HMW-secalins, γ-75k-secalins, and γ-40k-secalins in rye and β-hordeins, C-hordeins, D-hordeins, and γ-hordeins in barley, can be classified into three different groups according to their homologous (gliadins) amino acid sequences and MWs in the LMW group, medium-molecular-weight group, and HMW group. The amino acid sequences of these proteins feature repetitive motifs—such as QQPGQG, YYPTSP, or short repeats like QQP and QPG. Variations between these proteins and wheat gliadins often involve single amino acid substitutions or rearrangements of these repetitive units along the polypeptide chain, resulting in differences in their MWs. For example, the repetitive motifs for *ω*1,2-gliadins (QPQQPFP) are characteristic both of ω-secalins and C-hordeins. The MWs of LMW proteins—which include monomeric proteins such as α-gliadins, γ-gliadins, γ-40k-secalins, and γ-hordeins, as well as polymeric proteins like LMW glutenin subunits (LMW-GS), γ-75k-secalins, and β-hordeins—generally range from 28 to 35 kDa. An exception is γ-75k-secalins, which exhibit a higher MW of approximately 50 kDa. These proteins are characterized by unique repetitive motifs, such as QQPQQPFP found in γ-75k-secalins and β-hordeins, and are also present in wheat γ-gliadins. These repetitive sequences are key contributors to CD immunoreactivity in wheat and, similarly, are implicated in the pathogenic potential of rye and barley, as most CD-active peptides originate from these conserved motifs [18,19,20]. In contrast, avenins in oats are present in much lower amounts (<2% of flour) and generally exhibit fewer and shorter repetitive motifs. While some avenin peptides can elicit immune responses in a subset of CD patients, their overall immunogenic potential is lower compared to wheat, rye, and barley prolamins, consistent with the lower prevalence of avenin-related celiac reactivity [2].

## 3. Gluten Toxicity: Health Disorders Associated with Gluten Ingestion

### 3.1. Pathogenesis of CD

CD is a complex autoimmune disorder triggered by the ingestion of gluten in genetically predisposed individuals who express specific human leukocyte antigen (HLA) class II haplotypes, namely HLA-DQ2 and HLA-DQ8. Two main conditions are necessary for the development of CD: the presence of a genetic predisposition and the consumption of gluten [21]. The primary environmental risk factor for CD is the ingestion of gluten and related prolamins, which possess strong immunodominant properties in genetically susceptible individuals. Upon ingestion, gluten proteins are only partially hydrolyzed by gastrointestinal proteases, resulting in the formation of peptides that are highly resistant to further enzymatic degradation. This resistance is primarily due to the unique amino acid composition of gluten proteins—especially their high content of proline and glutamine residues arranged in repetitive motifs. These structural features render gluten peptides resistant to cleavage by key human gastrointestinal enzymes, including pepsin, trypsin, chymotrypsin, carboxypeptidases A and B, elastases, and brush-border membrane enzymes of the small intestine [22]. Notably, these enzymes exhibit limited activity at peptide bonds adjacent to proline residues, allowing the persistence of relatively large peptides (often nine or more amino acids in length). These peptides can cross the intestinal epithelium via paracellular or transcellular pathways and accumulate in the lamina propria. Gluten-derived peptides, rich in glutamine, are a good substrate for tissue transglutaminase (TGase), an enzyme released during inflammation. TGase deamidates these peptides, increasing their affinity for HLA-DQ2 and HLA-DQ8 molecules expressed on antigen-presenting cells in the gut. Once bound to these HLA molecules, the deamidated gliadin peptides are presented to CD4+ T cells, triggering a gliadin-specific immune response. This immune activation leads to intestinal inflammation and subsequent tissue damage characteristic of CD [22,23].

Extensive studies have identified several disease-driven gluten T cells epitopes, mainly derived from wheat—particularly from α-, γ-, and ω-gliadins as well as from glutenins—and, to a lesser extent, from barley and rye through their specific prolamins, hordeins, and secalins [24,25,26]. Nevertheless, many gluten T cell epitopes remain unidentified. Immunogenic sequences were identified using different approaches, ranging from mass spectrometry analysis of peptide fragments [27,28,29] to tagging of glutamine residues by TGase [30,31] to homology searches in edible cereal proteins [32], or by detection of gluten-specific T cells in the peripheral blood of CD patients after short-term consumption of gluten-containing food [32,33]. All the peptides identified contain one or more epitope sequences recognized by HLA-DQ2 and HLA-DQ8 molecules [33] after TGase-mediated deamidation [8,10,22]. A detailed list of CD epitopes and their nomenclature has been compiled by Sollid et al. [25], while a comprehensive repertoire of gluten peptides recognized by celiac T cells has been reviewed by Camarca et al. [24].

Several studies have evaluated peptides resistant to gastrointestinal hydrolysis using in vitro digestion of gliadins or gluten-containing foods. These investigations led to the identification of immunogenic gastro-resistant peptides (GIP), which include one or more epitope sequences [34,35,36,37]. Among these, the most emblematic is the 33-mer peptide (LQLQPFPQPQLPYPQPQLPYPQPQLPYPQPQPF), located in the N-terminal region 57–89 of α-gliadins. This fragment contains six copies of three different T-cell epitopes (DQ2-α-I, DQ2-α-II, and DQ2-α-III) and is characterized by a remarkable resistance to gastrointestinal proteolytic digestion, which amplifies its immunogenic potential [22]. In γ-gliadins, by contrast, the distribution of immunogenic sequences is more heterogeneous, with numerous stimulatory epitopes spread throughout the protein sequence. Among them, the 26-mer peptide (FLQPQQPFPQQPQQPYPQQPQQPFPQ) is particularly relevant, along with several shorter fragments containing immunogenic motifs such as QQPFPQQPQ [37]. A distinct category of gliadin peptides comprises the so-called ‘innate peptides’, which are not recognized by CD4+ T cells but are able to elicit an innate immune response in antigen-presenting cells (APC) such as monocytes, macrophages, and dendritic cells. The most studied peptide in this category is the α-gliadin p31–43 (or p31–49) peptide [38]. This peptide, or fragments including this sequence, has been shown to be highly resistant to in vitro gastrointestinal hydrolysis [39,40]. More recently, in vivo studies have revealed its detection in urine, confirming its remarkable resistance to proteolysis, intestinal permeability, and systemic circulation up to renal excretion [41].

In genetically predisposed individuals, the consumption of gluten or gluten-related proteins can trigger an aberrant immune response that leads to the development of symptoms associated with CD [2]. However, additional susceptibility factors beyond genetics have also been implicated. Alterations in the intestinal microbiota—specifically, reduced microbial diversity and shifts in microbial composition—have been associated with an increased risk of CD. Furthermore, emerging evidence suggests that certain viral infections may act as environmental triggers, contributing to the onset of the disease [42]. Clinically, CD is characterized by a range of symptoms primarily associated with malabsorption, including chronic diarrhea, steatorrhea, fatigue, weight loss, anemia, dermatological manifestations, and, in children, growth failure [2]. There is no defined minimum threshold of gluten that causes adverse effects, as this depends on individual patients. However, the scientific community recognizes that most CD subjects can tolerate trace amounts below 20 ppm, which is also the regulatory threshold for labeling a food as gluten-free [43]. To date, the only effective treatment for CD is a lifelong adherence to a strict gluten-free diet, which typically leads to symptom resolution and mucosal healing [44].

### 3.2. Non-Celiac Gluten Sensitivity

Non-celiac gluten sensitivity is a wheat-related condition that is neither IgE-mediated and not associated with an autoimmune response [45]. Determining the prevalence of NCGS is challenging due to the absence of validated biomarkers. Moreover, since the offending components may include other wheat constituents beyond gluten—such as amylase–trypsin inhibitors and fermentable carbohydrates (FODMAPs)—many experts now favor the broader term “non-celiac wheat/gluten sensitivity” [21]. Amylase–trypsin inhibitors (ATIs) are highly digestion-resistant proteins that can activate innate immunity and amplify symptoms [46]. FODMAPs may also provoke gastrointestinal distress; in some patients, they seem to be the main trigger, as low-FODMAP diets often bring marked relief [21]. It has been demonstrated that ancient diploid wheat (*Triticum monococcum*) activates immune cells far less than modern tetraploid or hexaploid varieties, suggesting it may be better tolerated by NCGS sufferers. Likewise, *Tritordeum*—a modern hybrid of durum wheat and barley—contains relatively little immunogenic gliadin and has been proposed as another potentially safe alternative [47,48].

Clinical symptoms of NCGS are generally less severe than those observed in CD or IgE-mediated wheat allergy [21]. Common manifestations include abdominal pain, bloating, gas, and diarrhea. Although these symptoms can resemble those of CD or wheat allergy, individuals with NCGS do not exhibit IgA anti-tissue transglutaminase autoantibodies or specific IgE antibodies against wheat proteins. Unlike CD, NCGS does not usually cause permanent intestinal damage, and symptoms generally improve or resolve with a gluten-free diet or reduced gluten intake [49]. While a minimum safe gluten threshold has been established for CD patients, no comparable value can be defined for individuals affected by NCGS, as their reactions are highly variable and dose-dependent [50].

### 3.3. Wheat Allergy

Wheat allergy is an adverse immune response triggered by either IgE-mediated or non-IgE-mediated mechanisms. Two distinct types of IgE-mediated allergic diseases related to wheat and other cereals have been identified. The first type includes occupational respiratory allergies, such as baker’s asthma and rhinitis, which result from inhaling flour particles in environments like bakeries. The second type refers to a true IgE-mediated food allergy to cereals. This process begins with the sensitization phase, where the immune system produces IgE antibodies specific to wheat allergens. Upon subsequent exposure, these allergens (e.g., wheat proteins) bind to IgE antibodies on the surface of mast cells and basophils, causing them to activate. This activation leads to the release of pro-inflammatory mediators such as histamine, which are responsible for the clinical symptoms of allergy [51,52,53].

According to the Allergen Nomenclature Sub-Committee of the International Union of Immunological Societies (IUIS), 28 distinct wheat allergens have been identified to date [54]. These allergens are present in both the water-insoluble fraction—comprising gliadins and glutenins—and the salt-soluble fraction, which includes lipid transfer proteins (LTPs) and α-amylase inhibitors (ATIs). Both ATIs and LTPs, such as Tri a 14, are heat-stable and are frequently recognized by IgE antibodies in patients with severe reactions to wheat, including wheat-dependent exercise-induced anaphylaxis (WDEIA) and baker’s asthma. Another heat- and digestion-resistant allergen, Tri a 37, a plant defense protein, has also been associated with severe allergic responses to wheat. Additionally, ω5-gliadin, designated as Tri a 19, has been strongly implicated in WDEIA and anaphylactic reactions to wheat, particularly in children [55,56].

The clinical symptoms of wheat allergy vary widely. They may present as typical food allergy symptoms affecting the skin, gastrointestinal tract, or respiratory system. In rare cases, a severe condition known as WDEIA may occur. This form is marked by respiratory distress, urticaria, throat tightness, and a rapid drop in blood pressure, typically following wheat ingestion combined with physical activity [51,56]. In IgE-mediated wheat allergy, allergic reactions are dose-dependent and vary between individuals. The exact threshold depends on the patient’s sensitivity [50].

Currently, the primary approach to managing wheat allergy involves strict avoidance of wheat-containing products. There is, however, no validated strategy for primary prevention. Developing hypoallergenic wheat-based foods with reduced allergenic proteins may offer a promising preventive option and help lower the incidence of wheat allergies in the future.

### 3.4. Detection and Quantification of Gluten Peptides

For individuals with gluten-related disorders, gluten must be strictly avoided in the diet, including even trace amounts that may result from cross-contamination, which is particularly critical for CD patients. Cross-contamination can occur at various stages of the food supply chain, including harvesting, transportation, storage, and processing, when gluten-free grains come into contact with gluten-containing cereals. Additionally, shared equipment or facilities in food manufacturing further increase the risk of unintentional gluten contamination.

Given these challenges, the development and application of reliable methods for the detection and quantification of gluten peptides is of paramount importance. Different investigative techniques are employed to determine gluten peptides, including immunochemical-based methods (ELISA, lateral flow devices) and proteomics-based methods (mass spectrometry) [57]. These methods differ significantly in terms of cost, sensitivity, and specificity. Nevertheless, they must be sensitive enough to detect trace levels of gluten, specific enough to avoid false positives, and robust enough to be applied across a wide variety of food matrices [58].

ELISA is a widely used immunochemical method for the detection and quantification of gluten in raw and processed foods, as well as beverages. Its ease of use and rapid results make it the most common method for gluten analysis in the food industry. Commercial ELISA kits typically employ monoclonal antibodies such as R5 and G12 [57]. The Codex Alimentarius standard for gluten detection officially endorses the use of the R5 monoclonal antibody, which recognizes specific peptide epitopes (QQPFP, QQQFP, LQPFP, and QLPFP) present in α-/β-, ω-, and γ-gliadins, not only in wheat but also in rye (secalin) [59]. The G12 monoclonal antibody was developed to target the immunotoxic 33-mer peptide of α2-gliadin. G12 specifically binds to the QPQLPY epitope, one of the most immunogenic fragments of the 33-mer [60]. Novel monoclonal antibodies that specifically recognize deamidated gliadin have also been proposed for the detection of gluten proteins that may be modified during food processing [60,61].

Sandwich ELISA is the commonly used format for detecting gluten contamination from intact proteins, as it requires two distinct epitopes on the target molecule for antibody binding. However, it is not effective for detecting hydrolyzed gluten, where proteins are broken down and epitopes may be lost. In contrast, competitive ELISA is more suitable for detecting small antigens that possess only a single epitope, such as hydrolyzed gluten fragments. Competitive ELISAs based on R5 and G12 antibodies are commonly used for this purpose [62,63].

Immunochemical assays have also been developed not only for the detection of gluten in food but also for the analysis of gluten immunogenic peptides in biological fluids, in order to monitor dietary intake and gluten exposure. An ELISA based on G12 has been developed for the detection of trace gluten in feces [64]. Lateral flow devices are rapid, strip-based tests that detect prolamin epitopes to estimate gluten content, similar to ELISA methods, generally based on G12 and A1 antibodies [65,66]. They are commonly used for onsite testing within manufacturing facilities as part of control programs and to support hazard analysis and critical control point (HACCP) systems—from raw material storage to final products. LFDs offer key advantages, including user-friendliness, rapid results, and the convenience of on-site analysis [64,65,66,67].

Mass spectrometry-based proteomic analysis has been applied for the identification and quantification of immunoreactive cereal proteins [55] and is increasingly used as an alternative or complementary approach to immunochemical methods. This approach provides not only qualitative and quantitative results but also a detailed characterization of the amino acid sequences of gluten peptides [68,69,70].

These methods are based on the release of immunogenic peptides following enzymatic treatment, followed by their detection and quantification. Typical targets include α- and γ-gliadin-derived peptides, and often the proteolytically resistant 33-mer peptide [68,69,70], which is believed to play a pivotal role in the pathogenesis of CD [22,37,68,71]. Thanks to its versatility, this technique has been proposed for the analysis of gluten in foods as well as for the detection of gluten immunogenic peptides in biological samples. In particular, a recent study identified gluten peptides in subjects after the ingestion of gluten-containing foods. Urine peptidomic analyses revealed high variability in gluten peptides between subjects, which may be due to interindividual differences in protein digestion and gastrointestinal damage [41,72,73].

## 4. Beneficial Aspects of Gluten-(Derived Peptides)

Beyond their well-known role in the structure/functionality of gluten as well as their involvement in CD, wheat proteins are also a source of bioactive peptides with potential beneficial effects on human health [74,75]. These peptides are inactive within the native protein sequence but can be released through enzymatic hydrolysis, either during gastrointestinal digestion, food processing such as fermentation or germination, or through the action of microbial proteases [76,77]. Several bioactive peptides have been identified from wheat proteins, particularly from gliadins and glutenins, but also from non-gluten proteins like albumins and globulins, and they exhibit a wide range of physiological functions [78,79].

Among the most studied activities is the antioxidant potential of certain peptides capable of scavenging free radicals and inhibiting lipid peroxidation, thereby contributing to the reduction of oxidative stress—a key factor in the development of chronic inflammatory diseases. These antioxidant peptides are typically released from albumins and globulins following germination or fermentation and include sequences such as AREGETVVPG, short peptides like GPF, GPE, and FGE (with molecular weights < 1.5 kDa), as well as others like YDWPGGRN, TGP, and QPYPQQPQ, which may also exert protective effects against oxidative stress in intestinal cells such as Caco-2 and possibly display anti-aging properties [39]. In addition, several wheat oligopeptides such as LY, PY, YQ, APSY, RGGY, and LVS have demonstrated both antioxidant and antihypertensive activities, supporting their potential use in nutraceutical applications [80].

Another notable functional category includes peptides with angiotensin-converting enzyme (ACE) inhibitory activity, which may help reduce blood pressure by modulating the renin-angiotensin system. For instance, the tripeptide IAP, derived from gliadin hydrolysates, exhibits potent ACE-inhibition effects and has shown efficacy in lowering blood pressure in hypertensive rats. Other wheat-derived peptides, such as VPL, WL, and WP, also demonstrate ACE-inhibitory effects, largely due to their hydrophobic amino acid content, especially proline and tryptophan. Asoodeh et al. [81] identified sequences like IPALLKR and AQQLAAQLPRMCR from wheat gluten hydrolysates, showing that ACE-inhibitory activity is strongly influenced by peptide structure, particularly the presence of hydrophobic and aromatic residues such as tryptophan, tyrosine, phenylalanine, and proline in the C-terminal region. Similarly, studies conducted on isolated peptides from wheat gluten for their potential use as ACE inhibitors emphasize the importance of generating gluten hydrolysates to enhance their health-promoting properties—an approach that may be particularly relevant for celiac individuals, provided that the hydrolysates are rendered non-immunogenic [82].

In vivo studies have demonstrated that wheat peptides, administered as dietary supplements, exert significant hypotensive and antioxidant effects. Experiments conducted on hypertensive rat models showed that hydrolyzed wheat gluten proteins and certain natural peptides derived from wheat are able to safely reduce blood pressure by inhibiting angiotensin production. In addition, wheat peptides exhibited marked antioxidant properties [82].

Similarly, in vivo experiments on animal models demonstrated that a mixture of corn and wheat peptides can prevent the onset of diabetes in NOD (non-obese diabetic) mice [83]. Moreover, wheat peptides have also been shown to protect against gastric damage induced by NSAIDs (Non-Steroidal Anti-Inflammatory Drugs) in rats, by reducing the oxidative stress associated with these drugs [84].

Another fascinating class of wheat-derived peptides is represented by gluten exorphins—opioid-like peptides released from gliadins during enzymatic digestion—which are capable of binding to opioid receptors and may influence mood, appetite, and gastrointestinal motility. While their pharmacological activity has been well documented in vitro, their physiological relevance in vivo remains debated. Key examples include gluten exorphin A4 (QYYP), A5 (GYYPT), B4 (YGGW), B5 (YGGWL), C5 (YPISL), and gliadorphin-7 (YPQPQPF) [81,82]. To exert their activity, these peptides would need to cross the intestinal barrier, reach the systemic circulation, and remain intact long enough to access the central nervous system—a condition that has not yet been clearly demonstrated. In such cases, gluten exorphins may exert effects on the central nervous system, potentially influencing behavior, attention, and emotional regulation [85,86,87,88]. Some hypotheses suggest a possible link between gluten-derived exorphins and neurological or neurodevelopmental disorders, such as autism spectrum disorders or schizophrenia, although conclusive clinical evidence is still lacking [85,86,87,88,89,90]. Therefore, while gluten exorphins may have interesting pharmacological properties, their potential role in adverse physiological or neurological effects cannot be excluded and warrants further investigation.

In addition to their antioxidant, antihypertensive, and opioid-like activities, certain wheat-derived peptides have demonstrated immunomodulatory properties by influencing cytokine production [91]. Furthermore, there is emerging evidence supporting the hypocholesterolemic and antidiabetic potential of specific peptides that inhibit key enzymes involved in carbohydrate and lipid metabolism, such as α-glucosidase and pancreatic lipase, suggesting their potential usefulness in managing metabolic syndrome [92,93].

**Figure 1:**
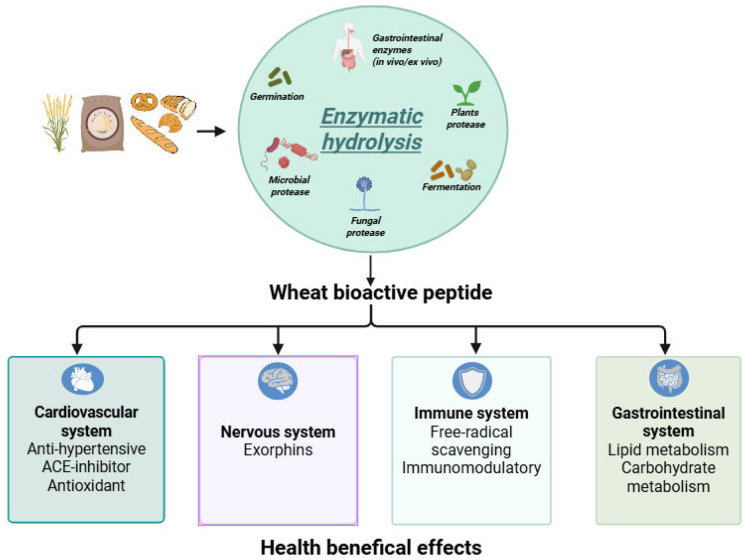
Potential biological activities of wheat-derived bioactive peptides released by enzymatic hydrolysis. The figure was created with BioRender: https://www.biorender.com/ (accessed on 2 September 2025).

It is important to note that the bioactivity and physiological relevance of these peptides depend largely on their bioavailability, particularly their resistance to gastrointestinal digestion and their ability to reach target tissues intact [94,95]. In this context, hydrolysis with selected enzymes has proven to be a promising strategy to enhance the release, stability, and functionality of bioactive peptides, especially in the development of functional wheat-based foods designed to support health beyond basic nutrition (Figure 1).

## 5. Strategies to Minimize Gluten Toxicity While Enhancing Health Benefits

While adherence to a strict, lifelong gluten-free diet remains the only effective treatment, alternative strategies, in order to mitigate adverse immune responses in susceptible individuals—such as reducing the gluten content in food and beverages—are also being explored [96,97,98,99].

**Figure 2:**
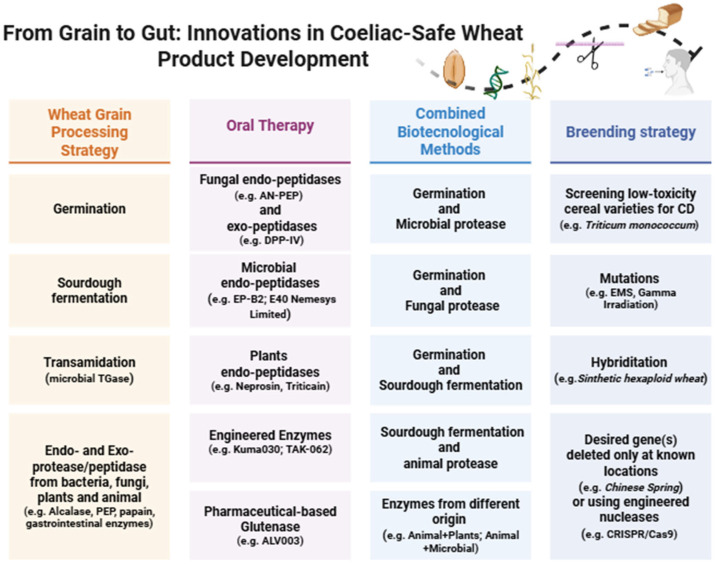
Biological and genetic strategies for the detoxification of wheat-based products. References [37,101,102,103,104,105,106,107,108,109,110,111,112,113,114,115,116,117,118,119,120,121,122,123,124,125,126,127,128]. Adapted and retrieved from BioRender templates: BioRender: https://www.biorender.com/ (accessed on 11 September 2025).

Over the last decade, several studies have focused on reducing or eliminating gluten toxicity by using methodologies based on biological measurements, genetic approaches, or a combination of biotechnological and non-biotechnological techniques (Figure 2). The objective was to apply non-invasive enzymatic strategies in order to hydrolyze immunogenic gluten peptides, while simultaneously enhancing, where feasible, the nutritional profile and organoleptic characteristics of the food matrix [100].

The use of proteolytic enzymes capable of degrading immunogenic peptide sequences rich in proline and glutamine is one of the paths to follow, thereby preventing the ingestion of peptides that elicit autoimmune responses in individuals with celiac disease. These enzymes, in fact, can be either exogenous—typically of microbial origin as lactic acid bacteria (LAB)—or endogenous, involved in natural metabolic processes during grain germination and sprouting. Endogenous activity may also include enzymes produced by microorganisms associated with the plant microbiome, such as fungal xylanases, which contribute to the breakdown of complex macromolecules during the early developmental stage. Table 1 provides a detailed summary of the degradation strategies implemented, the specific enzymes involved, and the conditions under which each method was applied.

### 5.1. Fermentation with Bacteria Strains

Fermentation with LAB is one of the most widely used biotechnological strategies to reduce the toxicity of gluten through microbial enzymatic activity. Numerous studies [143,144] have demonstrated that sourdough fermentation of wheat or gluten-containing flours reduces IgE recognition and that specific LAB starter cultures can partially degrade gluten proteins resistant to digestion. This enzymatic degradation may enhance gastrointestinal digestion and reduce sensitization risk by limiting exposure to intact immunogenic epitopes. However, achieving a truly gluten-free product using LAB cultures remains a significant challenge.

Pioneering work by Di Cagno et al. [131,132] identified the proteolytic capabilities of selected LAB strains—including *Lactobacillus alimentarius* 15M, *Lactobacillus brevis* 14G, *Lactobacillus sanfranciscensis* 7A, *Lactobacillus plantarum* CF1, and *Lactobacillus hilgardii* 51B—toward gliadin-derived peptides implicated in CD. These strains significantly degraded proline-rich sequences resistant to gastrointestinal proteases. Additionally, their use enhanced the nutritional quality of gluten-free bread formulations, demonstrating both detoxifying and nutritional benefits [101,133,145,146].

The application of probiotic LAB strains—such as *Streptococcus thermophilus*, *Lactobacillus plantarum*, *Lactobacillus acidophilus*, *Lactobacillus casei*, *Lactobacillus delbrueckii* subsp. *bulgaricus*, *Bifidobacterium breve*, *Bifidobacterium longum*, and *Bifidobacterium infantis*—have also been explored to reduce immunogenic gluten peptides in wheat dough. While these strains exhibit proteolytic activity, particularly during extended fermentation, they remain among the least effective strategies for achieving substantial gluten degradation [134,135,137].

Although sourdough-based fermentation does not lead to complete degradation of toxic gluten fragments, it contributes significantly to the improvement of texture, palatability, aroma development, and reduction of antinutritional factors in baked products [137].

Recently, *Lactococcus lactis* LLGKC18 was evaluated using a functional rat basophil leukemia (RBL) cell model activated with sera from allergic patients. The study showed significantly reduced RBL degranulation, indicating decreased allergenicity, despite the presence of residual peptides. MS/MS analysis confirmed the degradation of several immunogenic peptides, suggesting that *Lactococcus lactis* LLGKC18 produces proteases capable of targeting immunodominant sequences of *ω*5-gliadin [136].

Furthermore, various *Bacillus* species—including *Bacillus subtilis*, *Bacillus tequilensis*, and *Bacillus cereus* QAUSD07—have been shown to produce peptidases and proteases, such as subtilisin and thermolysin, that hydrolyze gliadin into smaller peptides and amino acids. Notably, proteases from *Bacillus subtilis* exhibit high specificity toward proline-rich regions, effectively degrading immunogenic peptides such as the 33-mer [102,147].

Recent advances in food processing technologies have increasingly targeted the elimination of toxic gluten epitopes through combined biotechnological strategies. One notable approach involves the use of fungal proteases in combination with probiotic *Lactobacillus* strains, particularly *Lactobacillus sanfranciscensis*, during extended sourdough fermentation [148]. This method has proven effective in vitro, achieving gluten degradation to levels below 10 ppm, which meets the threshold for gluten-free labeling and significantly reduces immunogenic potential [102,147,148].

A synergistic formulation of probiotic strains—including *Lactobacillus casei* LC130, *Lactobacillus paracasei* LPC100, and *Streptococcus thermophilus* ST250—has demonstrated the ability to hydrolyze immunoreactive gliadin peptides. This enzymatic activity holds promise for mitigating the effects of inadvertent gluten exposure, such as contamination in gluten-free foods, offering potential benefits for individuals with gluten-related disorders [134,135,138,148].

Additional evidence suggests that sourdough fermentation, combining yeast and LAB, further reduces gluten immunogenicity by promoting gluten depolymerization and hydrolysis, particularly through weakening glutenin polymerization and breaking down glutenin-derived peptides. Fermentations involving *Lactobacillus brevis* and *Pediococcus pentosaceus*, in combination with yeast, have shown a significant reduction of toxic gluten proteins in wholemeal products [134,135,138,148].

Importantly, the application of LAB in vivo-like settings has also been explored. A recent study by Nikoloudak et al. [149] evaluated a novel formulation combining probiotic strains with commercial proteolytic enzymes under simulated gastrointestinal conditions. The treatment successfully degraded gluten to below 20 ppm, the accepted safety threshold, indicating its strong potential for mitigating gluten-related immunotoxicity in clinical contexts.

### 5.2. Combined Enzymatic Strategies: Germination Meets Microbial and Fungal Enzyme Applications

The application of glutanase, particularly those derived from plants, fungi, and other microorganisms, and often associated with endogenous protease, has seen significant advancement due to their capacity to function both in vivo and ex vivo in effectively hydrolyzing gluten. This enzymatic activity facilitates the degradation of immunogenic epitopes that are degraded or completely destroyed, thereby reducing the toxicity associated with gluten peptides [73,129].

One of the principal mechanisms to obtain gluten hydrolysis is the activation of endogenous proteases in grain by germination. In barley, for example, this physiological process induces the activation of specific cysteine proteases, which are capable of hydrolyzing immunotoxic amino acid sequences within hordein, the predominant prolamin storage protein [129]. Recent studies have also examined wheat gluten degradation during germination, revealing that endoprotease activity begins around day 3 and reaches its peak by day 7. These findings suggest that germination under controlled conditions can effectively reduce gluten immunogenicity. However, extended germination increases amylase activity, which may lead to technological issues [103,104,130].

In addition to germination, other approaches have explored enzymatic modification of gluten using peptidases as transferases. One study demonstrated that binding methionine to gluten using α-chymotrypsin could alter its structure, offering a novel strategy for reducing its immunoreactivity [103].

Other approaches to obtain low content gluten products are to use other types of barley cysteine proteases, as the papain-like C1A family and the C13 family, also called legumains, that degrade completely epitopes eliciting CD [105,106]. These classes of endoproteases are also present in plants. Notably, papain, bromelain, and actinidin—classified as cysteine endoproteases—exhibit significant hydrolytic activity. Papain has been utilized in wheat gluten hydrolysates, a by-product of wheat starch production, effectively disrupting the gluten protein structure. Bromelain has been employed to produce hypoallergenic wheat flour, as it facilitates the cleavage of peptide bonds adjacent to proline residues, which are typically resistant to proteolytic degradation. Actinidin, naturally found in kiwifruit, papaya, and pineapple, enhances gluten digestibility by promoting proteolysis during gastrointestinal digestion, thereby improving protein bioavailability in the small intestine [107].

More generally, promising enzymatic approaches using plant proteases have been explored to reduce gluten allergenicity through targeted hydrolysis. Protease extracts purified from *Nigella sativa* effectively hydrolyze gluten and gliadin, suggesting their potential for detoxifying gliadin proteins. Similarly, neprosin, a protease derived from *Nepenthes pitcher* plants, has shown selective activity toward gliadin. Additionally, *Cuminum cyminum* (cumin) latex has been reported to enhance pepsin’s proteolytic activity; however, its application in gluten hydrolysis has not yet been evaluated. Another promising candidate is caricain, extracted from *Carica papaya latex*, which is able to detoxify gliadin in whole wheat flour, supporting its potential as a natural protease for gluten degradation [105,106,107]. Furthermore, the combination of a microbial protease (alcalase) with a plant protease (papain) demonstrated significantly greater effectiveness in degrading gliadin compared to the use of either enzyme alone in wheat flour. As a result, the resulting flour extract exhibited substantially reduced allergenic properties, outperforming hydrolysates produced using individual enzymes such as chymotrypsin, flavourzyme, trypsin, or pepsin [107].

Recent scientific studies increasingly employ a combination of endoproteases and exoproteases from different matrices to enhance the efficiency and specificity of gluten protein degradation [101]. The combination of a prolyl endopeptidase from *Flavobacterium meningosepticum* and a glutamine-specific endoproteinase from barley showed a detoxifying capacity of immunogenic gluten peptides during simulated gastrointestinal digestion. Another method is the synergistic employment of the prolyl endoproteases from *Aspergillus niger* (AN-PEP) and from *Sphingomonas capsulata* (SC-PEP), in conjunction with EP-B2, a cysteine endoprotease derived from barley, which has demonstrated enhanced efficacy in degrading proline-rich peptides, facilitating the breakdown of protease-resistant gluten peptides associated with CD pathogenesis. Furthermore, the cysteine endoprotease EP-B2 has also been extended to the brewing industry, employed to enzymatically detoxify hordeins, permitting the production of gluten-free beer from conventional cereals and pseudocereals while maintaining sensory and functional characteristics [108].

An innovative enzymatic approach for reducing the immunogenicity of gluten in CD involves the application of microbial transglutaminase (mTG), an endo-γ-glutamine:ε-lysine transferase. This enzyme catalyzes transamidation reactions, forming isopeptide bonds between the γ-carboxamide group of glutamine residues in gliadin and primary amines such as lysine or n-butylamine. Importantly, this targeted modification of gliadin peptides has been shown to prevent the formation of immunodominant T-cell epitopes, thereby inhibiting interferon-γ (IFN-γ) production by intestinal T cells from CD patients. These findings highlight transamidation as a promising technological intervention for developing wheat-based products with reduced immunogenic potential, offering a potential pathway toward safer dietary options for individuals with CD [109,110].

### 5.3. The Oral Therapy

Enzyme supplement therapy using either bacterial or fungal endopeptidases or endoproteases from germinating cereals has been suggested to promote the hydrolysis of gluten proteins and destroy toxic peptides. A prerequisite is that such enzymes are first safe for human consumption and then obviously active under gastro-duodenal conditions. Several enzymes, including prolyl endopeptidases, cysteine proteases, and subtilisins, can hydrolyze the human digestion-resistant gluten peptides both in vitro and in vivo [37].

EP-B2, a glutamine-specific endoprotease active during barley seed germination and commonly used in enzymatic treatments, remains active under gastric conditions (low pH), is resistant to pepsin, but is hydrolyzed at physiological concentrations of trypsin. It exhibits strong specificity for the QXP sequence, which is abundant in the immunotoxic 33-mer and other gluten-derived peptides. These properties make EP-B2 a promising candidate for use as a gastric-active enzyme in CD [111].

Proline endoproteases (PEPs) have been investigated as potential oral glutenases for CD therapy [112,113]. These serine proteases cleave peptide bonds at the carboxy end of proline residues, a common feature of immunogenic gluten peptides. Microbial sources such as *Flavobacterium meningosepticum* (FM-PEP), *Sphingomonas capsulata* (SC-PEP), and *Myxococcus xanthus* (MX-PEP) have been studied for this purpose [37]. Among them, SC-PEP shows the greatest promise due to its stability across acidic pH levels and resistance to pepsin degradation, unlike FM-PEP and MX-PEP, which are susceptible to digestion by pepsin and bile salts.

Another promising enzyme is a prolyl endopeptidase from the fungus *Aspergillus niger*, named AN-PEP, which is industrially scalable, active at gastric pH (3–5), and resistant to pepsin, similar to SC-PEP. Although less specific for immunodominant gluten epitopes, AN-PEP efficiently degrades gliadin into smaller peptides and may enhance gluten detoxification when used in combination with more selective enzymes, such as dipeptidyl peptidase-IV (DPP-IV) [23]. DPP-IV occurs naturally in small amounts in the small intestinal brush border; however, it has been obtained commercially from the fungus *Aspergillus oryzae*. It acts on the amino-terminal side to liberate X-Pro dipeptides of gliadin, and it works best at neutral pH; hence, it starts action only in the intestine, and it exploits a limited proteolytic effect [75,101].

Another fungal enzyme from *Aspergillus niger*, the aspergillopepsin (ASP), has demonstrated high efficiency in degrading CD–active peptides. With an optimal activity at pH 3.0, ASP can extensively hydrolyze dietary gluten into short peptides. However, it lacks specificity for the immunodominant 33-mer peptide of α2-gliadin. ASP is considered safe for human consumption and may be used in combination with more specific and potent gluten-degrading enzymes, such as EP-B2 or microbial prolyl endopeptidases, to enhance overall therapeutic efficacy [108,114].

A cysteine protease from the papain family, Triticain-α from *Triticum aestivum*, has been demonstrated to efficiently cleave α-, γ-, and ω-gliadin and glutenin, exhibiting an optimal enzymatic activity under acidic conditions (gastric phase). However, under intestinal conditions and in the presence of trypsin, this enzyme becomes susceptible to inactivation. Therefore, its functional activity is likely limited to the gastric phase of gluten digestion, prior to intestinal transit [115].

Recently, E40, a bacterial protease derived from *Actinoallomurus* sp. A8 has shown high efficacy in degrading the immunodominant 33-mer peptide and whole gliadin proteins, with optimal activity across a pH range of 3–6. Recombinant production of E40 using *Streptomycetes*—recognized as a safe protein source for human consumption—further supports its suitability for dietary management of gluten-related disorders. E40 is resistant to degradation by both pepsin and trypsin and retains activity in the acidic environment of the stomach. In simulated gastrointestinal conditions, E40 effectively digests gluten without releasing immunotoxic peptides, thereby preventing the downstream activation of the inflammatory cascade characteristic of CD. Its efficacy has also been validated in various food matrices, including beer, bread, and pasta. Notably, gluten levels in E40-treated samples consistently fell at or below the 20 ppm threshold required for gluten-free labeling; in fact, only trace amounts of immunogenic peptides derived from ω- and γ-gliadins were observed. For these reasons, E40 has been proposed as a promising candidate for oral enzymatic therapy aimed at managing gluten toxicity [116,117].

Again, Kuma030, an engineered endopeptidase derived from the microbial protease kumamolisin of *Alicyclobacillus sendaiensis*, has demonstrated the ability to degrade a broad range of immunogenic gliadin epitopes under gastric conditions (optimal activity at pH 4). Its enzymatic properties and substrate specificity highlight its potential as a promising candidate for the enzymatic treatment of CD [118,119].

While these enzymes have demonstrated glutenase activity under optimal conditions, it remains unclear whether they fully eliminate all immunogenic epitopes and prevent immune activation. Most have been tested only in small gluten challenge studies in patients on a gluten-free diet. Further clinical trials are needed to assess their efficacy and safety in real-world settings.

### 5.4. Genetic and Breeding Approaches

Another way to produce gluten-free wheat-based foods is by developing wheat varieties with fewer harmful CD epitopes that trigger CD. Achieving this requires a thorough understanding of the complexity of the wheat genome and the structure of the gene families encoding gliadin proteins that harbor CD epitopes. At the genomic level, the presence of CD epitopes varies among gliadin genes, across the homoeologous wheat chromosomes (A, B, and D), and among different wheat varieties and species. The α-gliadins encoded by the D genome contribute the highest number of CD epitopes, while those from the B genome contribute the fewest. Similarly, for γ-gliadins, the greatest number of CD epitopes has been identified in genes located on the D genome. Although less sequence information is available for ω-gliadins, their role in CD immunogenicity has recently been recognized due to T-cell cross-reactivity with related epitopes in rye and barley.

Genetic variations naturally present in different wheat cultivars offer opportunities to develop varieties that are safe for individuals with CD. Particular attention has been given to *Triticum monococcum* (einkorn), an ancient diploid wheat species regarded as a promising candidate for a species with reduced content of CD epitopes and with more digestible wheat gliadin [120,140,141]. Unlike modern wheat, einkorn lacks the D genome that encodes the highly immunogenic 33-mer gliadin peptide. Multiple in vitro and ex vivo studies have shown that einkorn exhibits a significantly lower potential to provoke immune responses in individuals with CD [120]. Although einkorn proteins still contain some immunogenic and toxic peptides, subtle variations in their amino acid sequences may reduce their harmful effects, suggesting a potentially lower overall toxicity compared to modern wheat [142].

Recent advances in biotechnology, particularly RNA interference (RNAi), have enabled the development of low-gluten wheat varieties. RNAi works by introducing complementary RNA molecules that bind to the target mRNA, promoting its degradation and thereby preventing the translation of specific genes, reducing gliadin expression and developing wheat lines with fewer gluten genes and/or gluten genes with inactivated CD epitopes [121,139].

Recently, targeted gene editing using CRISPR/Cas9 has been applied to gliadins. These methods produce offspring with silenced, deleted, and/or edited gliadins, which, overall, may reduce the exposure of patients to CD epitopes. The application of CRISPR-Cas9 silencing technology was also used to silence CM3 and CM16 ATI genes in durum wheat, in order to obtain transgenic lines that showed an effective decrease in the target gene coding for allergenic proteins [122,123,124].

Synthetic hexaploid wheat breeding represents a promising strategy for developing *Triticum* species with reduced gluten immunogenicity. This approach involves hybridizing *Triticum turgidum* ssp. *durum* with *Aegilops tauschii* lines with low levels of CD-toxic gliadins. The resulting SHW lines could combine low gluten immunogenicity with valuable agronomic traits. These lines would serve as key resources in pre-breeding programs, facilitating the development of commercial wheat cultivars that are both agronomically competitive and less immunoreactive for individuals with CD or NCGS [125].

Deletion lines of wheat, such as those derived from “Chinese Spring,” have been used to remove specific chromosomal regions, targeting both CD-epitopes and functional traits like dough quality. For example, deletion of the 6DS arm, which contains the 6D α-gliadin locus, significantly reduced Glia-α1 and Glia-α3 immunogenicity but resulted in stiffer, less elastic dough. In contrast, removing ω-gliadins, γ-gliadins, and LMW-glutenins from chromosome 1DS lowered certain CD epitopes without compromising dough properties. However, these modifications involve genetic alterations that persist in the wheat lines, limiting their commercial viability in regions with strict regulations on genetically modified organisms (GMOs) [126].

Recently, chemical strategies for reducing gluten toxicity have been applied. The methane sulfonate, for example, induces G/C to A/T nucleotides in the DNA transitions, often causing missense mutations in α- and γ-gliadin genes, including within CD epitopes [127]. Gamma irradiation, in contrast, causes DNA strand breaks, leading to deletions, substitutions, or insertions. This can disrupt or delete gliadin genes and associated epitopes [128].

In the future, low gluten immunogenicity may become one of several traits prioritized in commercial wheat programs. In order to produce coeliac-safe food products will require significant effort and likely necessitate adaptations in industrial grain and food processing methods. Summarizing, two complementary approaches may contribute to this goal: plant breeding, to develop wheat varieties with a reduced immunogenic gluten fraction, and food-processing innovations that modify or remove immunogenic gluten components during grain and product processing.

## 6. Conclusions and Future Perspectives

The bioavailability and in vivo effects of gluten-derived bioactive peptides represent a promising but still underexplored field of research with important implications for human health. While numerous in vitro studies have demonstrated that these peptides possess bioactive properties, it remains crucial to deepen our understanding of their bioavailability and biodisponibility through well-designed animal models and human clinical trials. Such studies will help clarify the real health benefits of these bioactive peptides and support their potential use as functional food components or nutraceuticals.

In the context of CD, a strict gluten-free diet remains the only effective and widely accepted therapeutic approach. Despite this, innovative strategies aimed at reducing gluten toxicity are gaining traction, holding great promise in improving the quality of life for genetically predisposed individuals and may eventually offer alternatives or adjuncts to the gluten-free diet.

Similar therapeutic avenues are being explored for NCGS, although this condition’s pathophysiology remains less understood. The identification of the specific gluten-derived or other wheat-related molecules responsible for triggering symptoms in NCGS is an ongoing area of research, and future studies are needed to define precise diagnostic biomarkers and targeted therapies.

Ultimately, gluten should not be viewed as inherently harmful for the general population, but rather as a dietary component contraindicated only for individuals with specific genetic predispositions or sensitivities. For the majority, gluten-containing foods can be part of a balanced and nutritious diet, potentially offering beneficial bioactive peptides that support health. The challenge for future research will be to unravel the complex dual nature of gluten proteins—harnessing their positive effects while minimizing risks for susceptible individuals—thereby guiding personalized nutritional recommendations and innovative therapeutic strategies.

## Figures and Tables

**Table 1:** Overview of enzymatic, microbial, genetic, and oral therapeutic strategies for gluten detoxification.

Strategy Type	Process or Method/	Enzyme/Microbial Strain/Gene/Compound	Experimental Conditions/ Application Method	Mechanism of Action/Impact on Gluten	References
**Enzymatic** **degradation**	Germination	Endogenous enzymes of wheat grains	8 days (25 °C, 100% humidity)	Reduction of peptides eliciting immune response.	[101,129]
		Endogenous enzymes of wheat grains	7 days (20 °C, pH 5.5, 100% humidity)	Degradation of gluten (<LOD).	[130]
		Cysteine protease from barley germination (EP-B2)	36 h, 100% humidity	Hydrolysis of immunotoxic sequences.	[104]
**Microbial** **fermentation**	Fermentation with LAB	*Lactobacillus* species: *alimentarius* 15M, *brevis* 14G, *sanfranciscensis* 7A, *plantarum* CF1, *and hilgardii* 51B	24 h at 37 °C	Gluten degradation.	[131,132]
		*Streptococcus thermophilus*, *Lactobacillus. plantarum*, *Lactobacillus acidophilus*, *Lactobacillus casei*, *Lactobacillus delbrueckii* subsp. *Bulgaricus*	72 h at 30 °C under anaerobic conditions	Gluten degradation.	[101,133]
		*Bifidobacterium* species: *bifidum*, *longum*, *breve*, *animalis* and *infantis*	24 h at 30 °C under anaerobic conditions	Proteolysis of gluten proteins.	[134]
		*Lactobacillus. plantarum species*	24 h at 30 °C	Hydrolysis and solubilization of wheat proteins.	[135]
		*Lactobacillus. sanfranciscensis*	24 h at 30 °C	Gluten degradation.	[102]
		*Lactococcus lactis* LLGKC18	24 h at 37 °C	Degradation of immunodominant sequences of ω-5 gliadin	[136]
		Combination of *Lactobacillus casei* LC130 and *Lactobacillus paracasei* LPC100 with the *Streptococcus thermophilus*	2 h at 37 °C	Hydrolyzation of immunoreactive gliadin peptides.	[137]
		Combination of *Lactobacillus. brevis* and *Pediococcus pentosaceus*	24 h at 35 °C	Reduced toxic gluten peptides content.	[138]
		*Bacillus species: stearothermophilus, subtilis, tequilensis, licheniformis, thermoproteolyticus and cereus* QAUSD07	1 h at 70 °C pH 8 or 24 h at 37 °C	Degradation of immunogenic peptides such as the 33-mer.	[102,134]
	Fermentation with endo- and exoprotease/peptidase from bacteria, fungi, plants and animals	Proline endoproteases (PEP) from *Flavobacterium meningosepticum*, *Sphingomonas capsulata*, and *Myxococcus xanthus*	24 h at 37 °C	Reduction of immunoreactive gliadin peptides.	[112,113,136]
		Alcalase	1 h at 50 °C	Reduced the antigenicity of wheat gluten hydrolysates.	[107]
		Plants protease from *Nigella Sativa, Nepenthes pitcher* (Neprosis), *Carica papaya latex* (Caricain)	2 h at 37 °C; 80 min at 37 °C	Hydrolysis of gluten proteins.	[106]
		Plants protease as papain, bromelain and actinidin	1 h at 65 °C or 70 °C	Hydrolysis of gluten proteins.	[105,106,107]
	Transamidation	Microbial transglutaminase (mTG)	mTG catalyses the transamidation reaction that leads to the formation of an isopeptide bond.	Prevention of the formation of immunodominant gluten peptides.	[109,110]
**Oral therapy**	Enzyme supplement therapy using either bacterial or fungal endopeptidases or endoproteases	Proline endoproteases (PEP) from *Flavobacterium meningosepticum*, *Sphingomonas capsulata*, and *Myxococcus xanthus*.	Optimal activity pH range of 3–5 (gastric phase)	Efficiently degrades the most immune-toxic gluten.	[37]
		Kuma 030: engineered endopeptidase derived from the microbial protease of *Alicyclobacillus sendaiensis* (kumamolisin)	Optimal activity pH 4 (gastric phase)	Efficiently degrades the most immune-toxic gluten.	[118]
		TAK-062: engineered endopeptidase from the precursor Kuma030	Optimal activity pH 2.5–6.0 (both gastric and intestinal phase)	Degrade > 99% of gluten in a complex study meal in the human stomach and in vitro.	[119]
		Aspergillopepsin (ASP) from *Aspergirllus niger*	Optimal activity pH 3 (gastric phase)	Hydrolyzation of immunoreactive gliadin peptides. Often used in combination with as EP-B2 or microbial prolyl endopeptidases to enhance the therapeutic efficacy.	[117]
		DPP-IV from *Aspergillus oryzae*	Optimal activity pH 7 (intestinal phase)	Limited proteolytic effect alone. Often used in combination with PEP.	[75]
		Cysteine protease from wheat germination (Triticain-α)	Optimal activity pH 3–6 (gastric phase)	Hydrolysis of immunotoxic sequences of α-, γ-, ω-gliadin, and glutenin.	[115]
		E40 glutenase from Actinomycete strain *Actinoallomurus A8*	Optimal activity pH range of 3–6 (gastric phase)	Degradation of the immunodominant 33-mer peptide and whole gliadin proteins.	[116,117]
**Genetic** **modification**	CRISPR/Cas9	Mutations of Gli-γ1-1D, Gli-γ2-1B,γ-gliadins, α-gliadins	Gene-editing tool that uses a special protein (Cas9) to cut DNA at exact spots in order to add, remove, or change specific genes.	Removing or reducing the toxic fractions of gluten.	[122,123,124]
	Chinese Spring	Deletion of the 6DS arm	Remove specific chromosomal regions	Strong reduction of gliadin immunogenicity.	[126]
	Gamma irradiation	Deletions in gliadins genes	It cause DNA strand breaks, which may lead to genetic alterations such as deletions, substitutions, or insertions.	Removing or reducing the toxic fractions of gluten.	[128]
	RNAi	Translation in gliadins genes	It cause target mRNA degradation, which may lead to genetic alterations	Reducing gliadin expression and developing wheat lines with fewer gluten genes and/or gluten genes with inactivated CD-epitopes.	[139]
	Methane sulfonate	Mutations in α- and γ-gliadin genes	G/C to A/T nucleotides in the DNA transition	Removing or reducing the toxic fractions of gluten.	[127]
**Plant** **breeding**	Less immunogenic alternative crops	*Triticum monoccoum*	Ancient wheat varieties that lack the D genome, known to encode highly immunogenic peptides.	Includes significantly fewer toxic sequences.	[120,140,141,142]
		Synthetic hexaploid wheat	Hybridization of *Triticum turgidum ssp.* durum with *Aegilops tauschii* lines	Low levels of CD-toxic gliadins.	[125]

## Data Availability

No new data were created or analyzed in this study.

## Abbreviations

The following abbreviations are used in this manuscript:

APC	Antigen presenting cells
ATI	Amylase–trypsin inhibitors
CD	Celiac disease
FODMAP	Fermentable oligosaccharides, disaccharides, monosaccharides, and polyols
GMO	Genetically modified organism
IFN-γ	interferon-γ
kDa	Kilo Dalton
LAB	Lactic acid bacteria
LTP	Lipid transfer protein
MW	Molecular weight
NCGS	Non celiac gluten sensitivity
NSAIDs	Non-Steroidal Anti-Inflammatory Drugs
NOD	Non-obese diabetic
pI	Isoelectric point
RBL	Rat basophil leukemia
SDS-PAGE	Sodium Dodecyl Sulphate—PolyAcrylamide Gel Electrophoresis
WDEIA	Wheat-dependent exercise-induced anaphylaxis
